# Flood Stress as a Technique to Assess Preventive Insecticide and Fungicide Treatments for Protecting Trees against Ambrosia Beetles

**DOI:** 10.3390/insects7030040

**Published:** 2016-08-18

**Authors:** Christopher M. Ranger, Peter B. Schultz, Michael E. Reding, Steven D. Frank, Debra E. Palmquist

**Affiliations:** 1 Horticultural Insects Research Lab, USDA-Agricultural Research Service, 1680 Madison Ave., Wooster, OH 44691, USA; mike.reding@ars.usda.gov; 2 Department of Entomology, Ohio Agricultural Research and Development Center, The Ohio State University, 1680 Madison Ave., Wooster, OH 44691, USA; 3 Hampton Roads Agricultural Research and Extension Center, Virginia Polytechnic Institute and State University, Virginia Beach, VA 23455, USA; schultzp@vt.edu; 4 Department of Entomology, North Carolina State University, Campus Box 7613, Raleigh, NC 27965, USA; sdfrank@ncsu.edu; 5 USDA-Agricultural Research Service-Midwest Area, 1815 N. University St., Peoria, IL 61604, USA; deb.palmquist@ars.usda.gov

**Keywords:** *Xylosandrus germanus*, flood stress, Scolytinae

## Abstract

Ambrosia beetles tunnel into the heartwood of trees where they cultivate and feed upon a symbiotic fungus. We assessed the effectiveness of flood stress for making *Cercis canadensis* L. and *Cornus florida* L. trees attractive to attack as part of insecticide and fungicide efficacy trials conducted in Ohio and Virginia. Since female ambrosia beetles will not begin ovipositing until their symbiotic fungus is established within the host, we also assessed pre-treatment of trees with permethrin, azoxystrobin, and potassium phosphite on fungal establishment and beetle colonization success. Permethrin reduced attacks on flooded trees, yet no attacks occurred on any of the non-flooded trees. Fewer galleries created within flooded trees pre-treated with permethrin, azoxystrobin, and potassium phosphite contained the purported symbiotic fungus; foundress’ eggs were only detected in flooded but untreated trees. While pre-treatment with permethrin, azoxystrobin, and potassium phosphite can disrupt colonization success, maintaining tree health continues to be the most effective and sustainable management strategy.

## 1. Introduction

Non-native ambrosia beetles are highly destructive pests of trees growing in ornamental nurseries and tree fruit orchards [1,2,3]. *Xylosandrus germanus* (Blandford), *Xylosandrus crassiusculus* (Motschulsky), and *Xylosandrus compactus* (Eichhoff) are especially problematic [3,4]. More than 200 plant species are hosts for *X. germanus* and *X. compactus* and over 120 species for *X. crassiusculus* [3,4,5,6]. Adult females tunnel into the xylem and pith of trees, where they create chambers used for cultivating their symbiotic fungus and rearing offspring. Larvae and adults of *X. germanus*, *X. crassiusculus*, and *X. compactus* feed on *Ambrosiella grosmanniae* Mayers, McNew and Harr., *Ambrosiella roeperi* Harr. and McNew, and *Fusarium solani* (Mart.) Sacc., respectively [7,8,9]. A dark mycelial form of the symbiotic fungi can grow in the beetles’ absence, but a white ambrosial form consisting of conidia and sprout cells is only produced and maintained in association with the beetles [3,7,8,9,10,11,12,13]. Hosts can also become infected with a variety of secondary microorganisms, including some highly destructive plant pathogens [14,15,16].

Conventional insecticides are preventively applied to protect trees from attack, but no insecticide treatment provides 100% protection [3]. In some instances, efficacy studies have been hampered by an inability to ensure ambrosia beetle pressure on experimental trees, particularly untreated controls. Since ethanol is an important attractant used by many ambrosia beetles for locating vulnerable trees, stem injections of ethanol have ensured tree attractiveness to beetles [17,18] and thereby aided in assessing the efficacy of conventional insecticides [19,20], botanical formulations [21], and an anti-aggregation pheromone [22]. 

Recent studies determined that flooding of intolerant tree species naturally induces ethanol emission *in vivo* and attacks by *X. germanus*, *X. crassiusculus*, and *X. compactus* [23,24]. Our current study sought to assess flooding as an alternative to ethanol injection as part of efficacy trials. Previous studies have demonstrated that foundress females do not begin ovipositing until their symbiotic fungus is growing within the host galleries and consumption of the symbiotic fungus is necessary for larval development, pupation, and sexual development [10,11,12,13]. This dependence represents a biological weakness that could potentially be exploited for management purposes. Thus, our current study sought to assess the effects of pre-treatment with an insecticide (permethrin) and two fungicides (azoxystrobin and potassium phosphite). The specific objectives of our current study were to: (1) assess flood stress as a tactic for inducing ambrosia beetle pressure on trees; and (2) evaluate the impact of preventive insecticide and fungicide applications on establishment of the symbiotic fungus and ambrosia beetle colonization success. 

## 2. Materials and Methods

### 2.1. Experimental Trees and Imposing Flood-Stress

Container-grown eastern redbud *Cercis canadensis* L. trees were used for insecticide efficacy experiments conducted in Ohio and flowering dogwood *Cornus florida* L. trees were used in Virginia. Trees used in Ohio were approximately 5 years old, 2.1 m tall, and growing in 23.2 L containers (Haviland Plastics HHP Large Blow Molded Nursery Container; HC7; Haviland, OH, USA) with a mixture of aged pine bark, peat, and coarse sand (60:30:10 v:v:v). Trees used in Virginia were approximately 6 years old, 2 m tall, and growing in 26.5 L containers with composted pine bark:sand (8:1 v:v; Blow Molded Nursery Container; Nursery Supplies, Chambersburg, PA, USA).

Flood stress conditions were imposed using a pot-in-pot system based on Ranger et al. [23]. In short, a 40.4 L pot (Haviland Plastics HHP; HC10) was first lined with a plastic waste bag of 3 mil (0.076 mm) thickness. A 23.2 L pot (Haviland Plastics HHP; HC10) containing a single *C. canadensis* tree was then placed within the plastic lined pot. Flood stress was imposed by irrigating the media within the internal pot until there was standing water around the base of the tree. Flooded trees were checked daily to ensure standing water was maintained throughout the duration of experiments. Excess plastic liner surrounding the edge of the flooded pot was twisted and then tucked in between the two pots to prevent beetles from landing in the standing water. The same approach was used for flood stressing trees deployed in Virginia including 25.6 L and 56.8 L inner and outer pots, respectively, and a 3 mil. plastic bag.

### 2.2. Effects of Preventive Insecticide Treatment on Preference and Colonization Success

The non-systemic insecticide Perm-Up^®^ 3.2 EC (36.8% permethrin) was selected for preventive treatment experiments conducted in Ohio and Virginia. In Ohio, a Crown Spra-Tool Spray Gun (Crown Industrial Products Co., Hebron, IL, USA) with a snap-on head and standard nozzle attachment was used to apply the diluted insecticide solution until runoff (~15 mL per tree) to the entire stem at a rate of 4.73 L per 379 L. In Virginia, diluted insecticide solutions were applied to stems until runoff using a 2 L CO_2_ backpack sprayer with a one-nozzle boom at 40 psi (R&D Sprayers, Opelousas, LA, USA).

After drying, trees in Ohio were arranged in six randomized complete blocks within a mixed deciduous woodlot (40°47′3.13″N; 81°50′6.21″W). Trees within each block were 3 m apart and replicated blocks were separated by 6 m. Treatments tested in Ohio included: (1) flooded; (2) flooded plus permethrin; (3) non-flooded; and (4) non-flooded plus permethrin (*n* = 6 trees per treatment). Trees in Virginia were arranged in six randomized complete blocks along the edge of a mixed deciduous woodlot (36°53′49.08″N; 75°59′37.02″W). Treatments tested in Virginia included: (1) flooded; (2) flooded plus permethrin; and (3) non-flooded.

Flooding, treatment with insecticide, and deployment under field conditions occurred on 9-July-2014 in Ohio. Flood stress was maintained throughout the duration of the experiment until 27-July-2014. Trees deployed in Ohio were thoroughly examined for new galleries at 2, 5, 8, 15, and 18 days after treatment. Gallery entrances were circled with a waterproof marker (Sharpie^®^, Oak Brook, IL, USA) and quantified during each tree inspection. Flooding, treatment with insecticide, and deployment under field conditions in Virginia occurred on 6-May-2013. Trees were held under field conditions in Virginia until 21-May-2013. Trees were examined for new galleries at 3, 6, 9, 12, and 15 days after treatment. 

Trees deployed in Ohio that sustained attacks were also cut at the base 18 days after deployment and transferred to a walk-in refrigerator held at 5 °C. Trees were dissected under laboratory conditions and the following parameters were noted based on Ranger et al. [3,24]: (1) presence of the white conidial form of the purported symbiotic fungus within the gallery; and (2) presence of eggs, larvae, pupae, and living or dead adult foundresses within the gallery. Identification of the white ambrosial form of the purported symbiotic fungus growing within host galleries was not confirmed as part of our current study, but previous efforts have documented the presence, growth, and ecology of these symbionts within such galleries [3,7,8,9,10,11,12,13,14,15,16]. Adult ambrosia beetle specimens recovered from host tissues were identified to species and quantified. 

### 2.3. Effects of Preventive Fungicide Treatment on Preference and Colonization Success

Container-grown *C. canadensis* were used for experiments conducted in Ohio and Virginia. Preventive treatment with the systemic fungicides Heritage^®^ 50 WP (50% azoxystrobin; 0.57 g in 1892 mL water; Syngenta Corp., Wilmington, DE, USA) and ProPhyt^®^ (54.5% potassium phosphite; 9.46 mL in 1892 mL of water; Helena Chemical Co., Collierville, TN, USA) were used in experiments conducted in both locations. Diluted fungicide solutions were applied to stems until runoff using the aforementioned methods for trees deployed in Ohio and Virginia. 

Trees deployed in Ohio were arranged as previously described in five randomized complete blocks within a mixed deciduous woodlot (40°47′3.54″N; 81°50′2.29″W). Similarly, trees deployed in Virginia were arranged as previously described in four randomized complete blocks along the edge of a mixed deciduous woodlot (36°46′84″N; 76°38′21.7″W). Treatments tested in Ohio and Virginia included: (1) flooded; (2) flooded plus azoxystrobin; (3) flooded plus potassium phosphite; (4) non-flooded; (5) non-flooded plus azoxystrobin; and (6) non-flooded plus potassium phosphite (*n* = 5 and *n* = 4 trees per treatment in Ohio and Virginia, respectively). Trees used in Ohio were flooded, treated, and deployed on 20-May-2015 and maintained under field conditions until 9-June-2015. Attacks were monitored as previously described at 1, 6, 9, 12, 14, 16, and 20 days after deployment in Ohio. Stems and galleries were dissected and evaluated as previously described. Trees used in Virginia were flooded, treated, and deployed on 20-April-2015 and maintained under field conditions until 12-May-2015. Attacks were monitored as previously described at 2, 8, 14, and 22 days after deployment in Virginia. Trees were cut at the base on the last day of the experiment, stored at 5°C, and then dissected to identify specimens in galleries.

### 2.4. Statistics

Count data associated with ambrosia beetle attacks on flooded trees were compared using a count regression procedure and a non-zero inflated model with a Poisson distribution (PROC COUNTREG; version 9.3; SAS Institute Inc., Cary, NC, USA). Non-flooded trees were not included in the analyses since no attacks occurred on any of the non-flooded trees deployed as part of insecticide and fungicide experiments conducted in Ohio and Virginia.

## 3. Results

### 3.1. Preventive Insecticide Effects on Preference and Colonization Success

**Figure 1:**
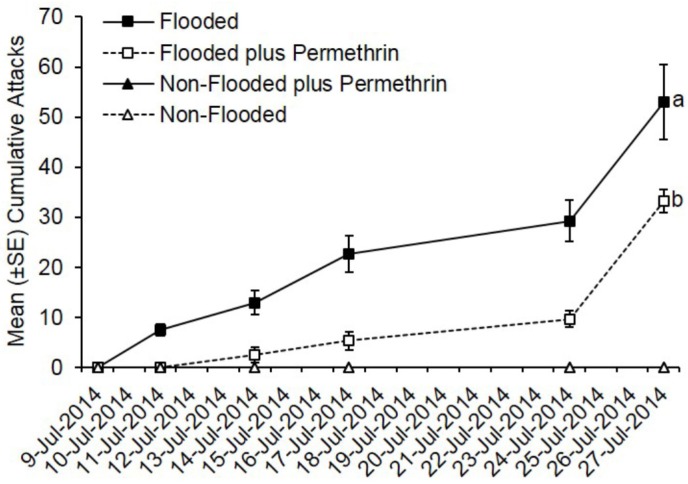
Impact of flood-stress and preventive insecticide treatment on ambrosia beetles attacks on *C. canadensis* trees deployed in Ohio. Trees were flood-stressed, treated with permethrin, and deployed on 9-July-2014. Means with different letters on 27-July-2014 are significantly different (flooded vs. flooded plus permethrin: *t* = −7.48; 1; *p* < 0.0001). Only the flooded trees were included as part of the analysis because no attacks occurred on any of the non-flooded trees.

Ambrosia beetles rapidly began attacking flooded *C. canadensis* trees deployed in Ohio with attacks being documented by 2 days after initiating flooding (Figure 1). In contrast, none of the non-flooded trees were attacked. Preventively treating flooded trees with permethrin significantly reduced the total number of cumulative attacks compared to flooded but untreated trees (*t* = −7.48; df = 1; *p* < 0.0001). A total of 427 attacks were associated with flooded but untreated trees compared to 232 attacks on trees that were flooded and treated with permethrin. A mean of 53.0 attacks occurred on flooded but untreated trees compared to 33.0 attacks on flooded trees pre-treated with permethrin.

Dissection of attacked trees revealed that significantly more ambrosia beetle galleries created in flooded but untreated trees contained the purported symbiotic fungus compared to flooded trees that were treated with permethrin (*t* = −5.04; df = 1; *p* < 0.0001) (Table 1). Furthermore, ambrosia beetle eggs were only found in galleries created within flooded but untreated trees; no eggs were observed in galleries created within flooded trees treated with permethrin (Table 1).

The non-native species *X. germanus* was the most abundant Scolytinae dissected from flooded *C. canadensis* trees deployed in Ohio; a total of 225 *X. germanus* were recovered from flooded but untreated trees compared to 111 specimens from flooded trees treated with permethrin (*t* = −6.03; df = 1; *p* < 0.0001) (Table 1). Significantly more living *X. germanus* foundresses were recovered from flooded but untreated trees compared to flooded trees preventively treated with permethrin (*t* = −3.03; df = 1; *p* = 0.003) (Table 1). One specimen of the non-native species *Anisandrus maiche* Stark was recovered from a flooded but untreated tree and also a flooded plus permethrin-treated tree. No native Scolytinae were recovered from any of the galleries created within the flooded trees (Table 1).

**Figure 2:**
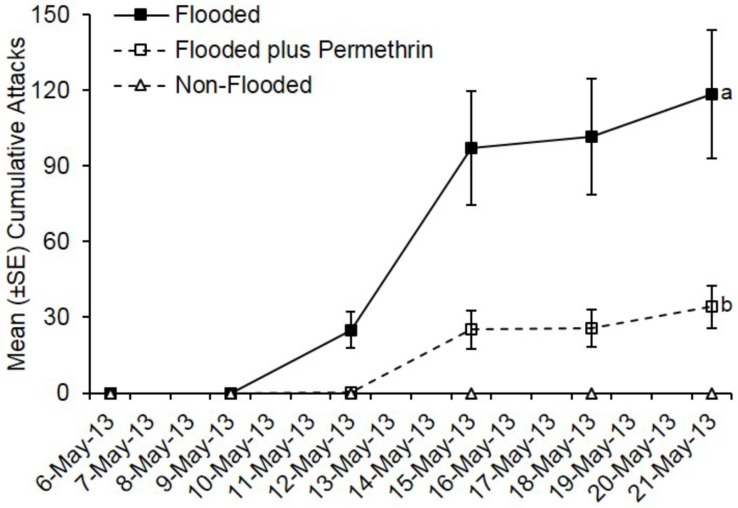
Impact of flood-stress and preventive insecticide treatment on ambrosia beetles attacks on *C. florida* trees deployed in Virginia. Trees were flood-stressed, treated with permethrin, and deployed on 6-May-2013. Means with different letters on 21-May-2013 are significantly different (flooded vs. flooded plus permethrin: *t* = −15.69; df = 1; *p* < 0.0001). Only the flooded trees were included as part of the analysis because no attacks occurred on any of the non-flooded trees.

Ambrosia beetles also preferentially attacked flooded *C. florida* trees deployed in Virginia, while none of the non-flooded trees were attacked (Figure 2). Pre-treatment with permethrin significantly reduced ambrosia beetle attacks on flooded *C. florida* trees deployed in Virginia (*t* = −15.69; df = 1; *p* < 0.0001). By day 15, a total of 711 attacks were associated with flooded but untreated trees compared to a total of 205 attacks on flooded plus permethrin-treated trees. A mean of 118.5 attacks occurred on flooded trees that were untreated compared with 34.2 attacks on flooded trees preventively treated with permethrin.

### 3.2. Preventive Fungicide Effects on Preference and Colonization Success

**Figure 3:**
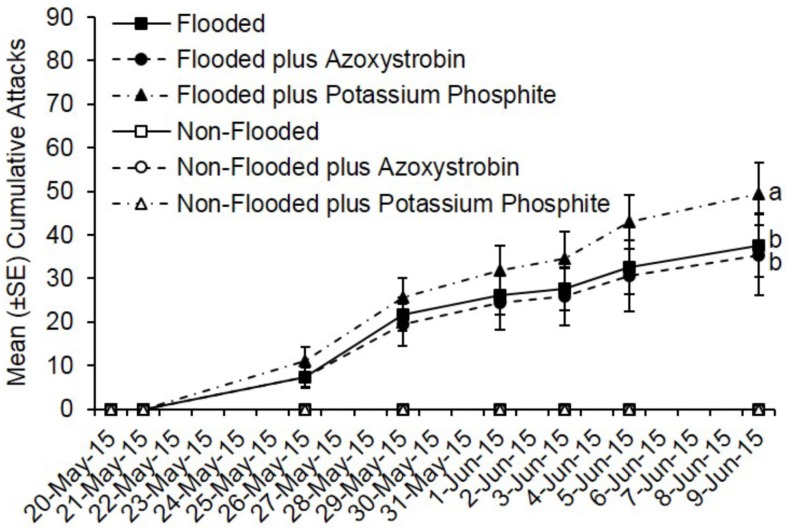
Cumulative ambrosia beetle attacks on *C. canadensis* trees deployed in Ohio that were either flooded or non-flooded and preventively treated with azoxystrobin, potassium phosphite, or untreated. Trees were flood-stressed, treated with selected fungicides, and deployed on 20-May-2015. Means with different letters on 9-June-2015 are significantly different (flooded vs. azoxystrobin: *t* = −0.58; df = 1; *p* = 0.57; flooded vs. potassium phosphite: *t* = 2.82; df = 1; *p* = 0.005). Only the flooded trees were included as part of the analysis because no attacks occurred on any of the non-flooded trees.

As with the insecticide trials, no ambrosia beetle attacks occurred on non-flooded *C. canadensis* trees deployed during fungicide trials conducted in Ohio and Virginia. Pre-treatment with azoxystrobin or potassium phosphite did not reduce ambrosia beetle attacks. Flooded trees deployed in Ohio were preferentially attacked, but by 20 days after pre-treatment, there was no difference in total attacks on flooded trees treated with azoxystrobin compared to flooded but untreated trees (*t* = −0.58; df = 1; *p* = 0.57) (Figure 3). However, more attacks occurred on flooded trees treated with potassium phosphite compared with flooded but untreated trees and flooded trees treated with azoxystrobin (*t* = 2.82; df = 1; *p* = 0.005). A total of 188 attacks occurred on flooded but untreated trees, 177 on flooded and azoxystrobin-treated trees, and 247 on flooded and potassium phosphite-treated trees (Figure 3). A mean of 37.6, 35.4, and 49.4 attacks occurred on flooded trees that were either untreated, treated with azoxystrobin, or treated with potassium phosphite, respectively.

Pre-treatment of flooded trees with azoxystrobin resulted in significantly fewer galleries with the purported symbiotic fungus compared to flooded but untreated trees (*t* = −4.77; df = 1; *p* < 0.0001) (Table 2). Trees pre-treated with potassium phosphite also had fewer galleries containing the purported symbiotic fungus compared to flooded but untreated trees (*t* = −3.38; df = 1; *p* = 0.0007) (Table 2). Galleries containing ambrosia beetle eggs only occurred in flooded *C. canadensis* trees that were untreated; no eggs were recovered from flooded trees pre-treated with azoxystrobin or potassium phosphite (Table 2).

*Xylosandrus germanus* was the most abundant Scolytinae species recovered from flooded trees deployed in Ohio; a total of 124 *X. germanus* were dissected from flooded but untreated trees, 108 specimens from flooded plus azoxystrobin-treated trees, and 157 specimens from potassium phosphite-treated trees (Table 2). There was no difference in the number of *X. germanus* dissected from flooded and untreated trees compared to flooded plus azoxystrobin-treated trees (*t* = −0.98; df = 1; *p* = 0.33), but more specimens were dissected from flooded trees pre-treated with potassium phosphite compared to untreated or azoxystrobin-treated trees (*t* = 2.02; df = 1; *p* = 0.04). However, more living *X. germanus* were recovered from flooded but untreated trees compared to azoxystrobin-treated trees (*t* = −5.08; df = 1; *p* < 0.0001) and potassium phosphite-treated trees (*t* = −3.16; df = 1; *p* = 0.002). The fewest living *X. germanus* were recovered from flooded trees treated with azoxystrobin. A single Scolytinae specimen, possibly *Micrasis swainei* Blackman, was recovered from a gallery created in a flooded tree treated with azoxystrobin and a flooded tree treated with potassium phosphite. 

**Figure 4:**
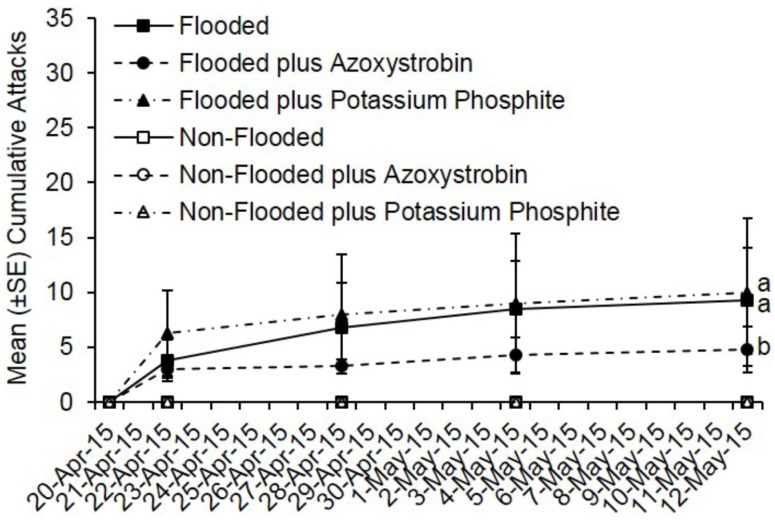
Cumulative ambrosia beetle attacks on *C. canadensis* trees deployed in Virginia that were either flooded or non-flooded and preventively treated with azoxystrobin, potassium phosphite, or untreated. Trees were flood-stressed, treated with selected fungicides, and deployed on 20-April-2015. Means with different letters on 12-May-2015 are significantly different (flooded vs. azoxystrobin: *t* = −2.36; df = 1; *p* = 0.02; flooded vs. potassium phosphite: *t* = 0.34; df = 1; *p* = 0.73).

By day 22, fewer total attacks occurred on flooded *C. canadensis* trees deployed in Virginia that were pre-treated with azoxystrobin compared to flooded but untreated trees and flooded plus potassium phosphite-treated trees (*t* = −2.36; df = 1; *p* = 0.018) (Figure 4). However, no difference was detected in total attacks on flooded but untreated trees compared to flooded plus potassium phosphite-treated trees (*t* = 0.34; df = 1; *p* = 0.73). A total of 37 attacks occurred on flooded but untreated trees, 19 attacks on flooded plus azoxystrobin-treated trees, and 40 attacks on potassium phosphite-treated trees. A mean of 9.3, 10.0, and 4.8 attacks occurred on flooded trees that were untreated, pre-treated with potassium phosphite, and pre-treated with azoxystrobin, respectively.

No eggs were observed in galleries created within flooded trees deployed in Virginia (Table 3). There was no difference in the number of larvae recovered from trees that were flooded and untreated compared to trees that were flooded and treated with potassium phosphite (*t* = −1.55; df = 1; *p* = 0.12); no larvae were recovered from flooded trees that were treated with azoxystrobin. Pupae were only recovered from flooded but untreated trees. *Xylosandrus germanus* was the only species dissected from flooded trees deployed in Virginia, but there was no difference among flooded trees that were untreated compared to those treated with azoxystrobin (*t* = −0.57; df = 1; *p* = 0.57) or potassium phosphite (*t* = 1.10; df = 1; *p* = 0.27).

## 4. Discussion

Flooding of container-grown *C. canadensis* and *C. florida* was an effective tactic for making trees naturally attractive to ambrosia beetles and thereby provided a basis for beetle pressure necessary for our efficacy experiments. Previous studies demonstrated that flooding induces ethanol production within stems of *C. canadensis* and *C. florida* [23,24]. Notably, all 21 flooded but untreated trees deployed in Ohio and Virginia (combined) were attacked by ambrosia beetles within a mean of 4 days after initiating flooding and they sustained a mean of 31.6 and 74.8 attacks per tree, respectively. None of the non-flooded trees deployed in Ohio and Virginia were attacked during any of the experiments. Pressurized stem injections of ethanol have been used to make trees attractive to ambrosia beetles for efficacy studies of conventional and botanically-based insecticides and repellents [19,20,21,22]. However, using flooding to naturally induce ethanol emission by experimental trees more accurately represents a stressed tree that a grower, arborist, or pest management professional would be treating under field conditions. Potential differences in the time-course emission of ethanol from flooded trees vs. ethanol-injected trees also makes flooding more biologically and ecologically relevant [23,25]. 

While injecting trees with ethanol is useful for creating highly attractive trap trees, we propose that flood stressing trees is an advantageous tactic for inducing ethanol emissions and making trees attractive to ambrosia beetles for efficacy studies. Yet, an important caveat for using flooding to induce ethanol emissions is that researchers must be careful to select tree species intolerant of flooding, such as *C. canadensis*, *C. florida*, and *Styrax japonicus* Sieb. et Zucc. [24]. Flood stress as a tactic for inducing attacks on trees as part of chemical control studies can also be labor intensive on a large scale compared to using excised bolts under laboratory conditions [26], but in some cases it can be advantageous to use intact living trees. Furthermore, it is unclear what impact flooding may have had on bark penetration, uptake, and efficacy of permethrin, azoxystrobin, and potassium phosphite by *C. canadensis* and *C. florida*. Permethrin is non-systemic, but azoxystrobin and potassium phosphite exhibit systemic activity [27,28]. However, since the products were applied as trunk sprays, it seems unlikely that bark penetration and localized systemic activity would have been negatively impacted.

Preventive application of permethrin to flooded *C. canadensis* and *C. florida* trees reduced but did not completely prevent attacks from occurring. Reding et al. [19] found permethrin was often associated with the fewest attacks on ethanol-injected trees compared to chlorantraniliprole, cyantraniliprole, tolfenpyrad, and dinotefuran, but pre-treatment did not provide 100% protection. Permethrin also minimized attacks on ethanol-injected trees and has been recommended for ambrosia beetle control, but repeated treatments may be required during peak ambrosia beetle flight [20]. In trials using treated stem sections (i.e., bolts), Mizell and Riddle [29] found cypermethrin and bifenthrin provided better control than permethrin, chlorpyrifos, and esfenvalerate, while acephae, cyfluthrin, endosulfan, fenpropathrin, imidacloprid, and thiamethoxam were ineffective. Frank and Bambara [30] also indicated chlorpyrifos was ineffective.

Minimizing or preventing attacks is optimal since ornamental producers essentially have a zero tolerance threshold due to high aesthetic standards and the potential for trees to be unmarketable or denied shipment by state horticulture inspectors [3]. However, disrupting colonization of the host is also an important component of ambrosia beetle management and can be especially beneficial for trees that sustained relatively few attacks. Furthermore, trees do not always die after attack and stems can heal if colonization is not successful. Since foundress females do not begin ovipositing until their symbiotic fungus is growing within the galleries [10,11,12,13], this phenomenon represents a vulnerability in their biology that can potentially be exploited for management purposes. Identifications of the purported fungal symbionts growing within host galleries of *C. canadensis* were not made as part of our current study. However, similar to previous observations [3,10,11,12,13], our current study confirmed that galleries were observed to contain the white conidial form of the purported symbiotic fungus in the absence of eggs, but no galleries were observed to contain eggs without the ambrosial form of the symbiotic fungus. Notably, the white conidial form of the symbiotic fungus is only produced and maintained in association with the beetles [10,11,12,13].

Permethrin, azoxystrobin, and potassium phosphite reduced the number of galleries created in flooded trees; permethrin and azoxystrobin also reduced the number of galleries containing the purported symbiotic fungus for trees deployed in Ohio. Furthermore, no eggs were found in galleries created in flooded trees that were pre-treated with permethrin, azoxystrobin, and potassium phosphite. Galleries containing the purported symbiotic fungus were not quantified for trees deployed in Virginia as part of the permethrin study; eggs, larvae, and pupae were not reduced by pre-treatment with either fungicide for trees deployed in Virginia. Previous insecticide efficacy studies generally focused on quantifying attacks and not establishment of the symbiotic fungus or presence of eggs, larvae, or pupae [19,20,21,22]. The mechanism by which permethrin pre-treatment reduced the number of galleries containing eggs and the purported symbiotic fungus is unknown but may be a result of increased beetle mortality or abandonment of galleries. 

We are not aware of previous studies that assessed the impact of fungicide pre-treatment on attacks, fungal establishment, or colonization success of *X. germanus* or other species of ambrosia beetles. However, Reddy and Verghese [31] recommended swabbing grape vines with a combination of the fungicide carbendazim and the insecticides dichlorvos and acephate as an effective treatment against *X. crassiusculus*. Furthermore, laboratory studies demonstrated the fungicides chlorothalonil, dimethomorph + mancozeb, propiconazole, and tebucozanole inhibited in vitro growth of the symbiotic fungus of *X. compactus* [32,33]. The direct or indirect mechanism by which azoxystrobin reduced the presence of fungal growth and ambrosia beetle eggs within host galleries of *C. canadensis* is not currently known, but could be related to systemic activity or gallery abandonment. 

## 5. Conclusions

Flooding represents an effective tactic for making *C. canadensis* and *C. florida* trees naturally attractive and vulnerable to attack by *X. germanus*. Our current study demonstrated preventive treatment with permethrin but not azoxystrobin or potassium phosphite reduced attacks by ambrosia beetles on flooded *C. canadensis*. While permethrin has been more effective than other conventional insecticides at minimizing attacks, it did not completely prevent them from occurring as part of our study and others. The low threshold for ambrosia beetle attacks on ornamental crops warrants the evaluation and identification of more effective chemical control options. Since fungal establishment and oviposition by foundress beetles can be disrupted by preventive treatment with permethrin and azoxystrobin, additional studies are warranted to determine if additive or synergistic effects occur with a combined pre-treatment of these active ingredients. Insecticides and fungicides are purportedly ineffective once beetles and their symbionts are established within their host tree [29,30], but reports of controlled studies are apparently absent in the literature. Based on our current study, additional studies are warranted to assess the efficacy of systemic insecticides and fungicides as rescue treatments for infested trees. However, the absence of attacks on any of the non-flooded trees deployed in Ohio and Virginia demonstrates that maintaining host vigor continues to be the most effective management strategy.

## Figures and Tables

**Table 1:** Influence of flooding and preventive insecticide treatment on attacks and colonization success of ambrosia beetles in *C. canadensis* deployed in Ohio.

Treatment	Mean (±SE) Number of			
	Galleries with Fungal Growth per Tree	Galleries with Eggs per Tree	Foundress *X. germanus* Recovered per Tree	Living *X. germanus* Recovered per Tree
Flooded	22.2 ± 5.5 ^a^	4.3 ± 1.7	37.5 ± 2.6 ^a^	3.2 ± 1.2 ^a^
Flooded plus Permethrin	10.2 ± 2.3 ^b^	0.0 ± 0.0	18.7 ± 3.2 ^b^	0.3 ± 0.2 ^b^
Non-Flooded	0.0 ± 0.0	0.0 ± 0.0	0.0 ± 0.0	0.0 ± 0.0
Non-Flooded plus Permethrin	0.0 ± 0.0	0.0 ± 0.0	0.0 ± 0.0	0.0 ± 0.0
*t*; *P*	−5.04; *p* < 0.0001	–	−6.03; <0.0001	−3.03; 0.003

Means with different letters within a column are significantly different using a count regression analysis with a Poisson distribution model (df = 1; *n* = 6 trees per treatment). Only the flooded trees were included as part of the analysis because no attacks occurred on any of the non-flooded trees. Number of eggs were not compared between flooded and flooded plus permethrin-treated trees because no eggs were associated with the later treatment.

**Table 2:** Influence of flooding and preventive fungicide treatment on attacks and colonization success of ambrosia beetles in *C. canadensis* deployed in Ohio.

Treatment	Mean (±SE) Number of			
	Galleries with Fungal Growth per Tree	Galleries with Eggs per Tree	Foundress *X. germanus* Recovered per Tree	Living *X. germanus* Recovered per Tree
Flooded	10.4 ± 3.8 ^a^	2.6 ± 1.9	24.8 ± 6.5 ^b^	13.8 ± 7.2 ^a^
Flooded plus Azoxystrobin	2.0 ± 1.0 ^b^	0.0 ± 0.0	21.8 ± 6.7 ^b^	3.6 ± 1.9 ^b^
Flooded plus Potassium Phosphite	4.4 ± 1.3 ^b^	0.0 ± 0.0	31.6 ± 4.9 ^a^	7.2 ± 2.2 ^b^
Non-Flooded	0.0 ± 0.0	0.0 ± 0.0	0.0 ± 0.0	0.0 ± 0.0
Non-Flooded plus Azoxystrobin	0.0 ± 0.0	0.0 ± 0.0	0.0 ± 0.0	0.0 ± 0.0
Non-Flooded plus Potassium Phosphite	0.0 ± 0.0	0.0 ± 0.0	0.0 ± 0.0	0.0 ± 0.0
*t*; *p*: Flooded vs. Flooded plus Azoxystrobin	−4.77; <0.0001	–	−0.98; 0.33	−5.08; <0.0001
*t*; *p*: Flooded vs. Flooded plus Potassium Phosphite	−3.38; 0.0007	–	2.02; 0.043	−3.16; 0.002

Means with different letters within a column are significantly different using a count regression analysis with a non-zero inflated model and Poisson distribution (df = 1; *n* = 5 trees per treatment). Only the flooded trees were included as part of the analysis because no attacks occurred on any of the non-flooded trees.

**Table 3:** Influence of flooding and preventive fungicide treatment on attacks and colonization success of ambrosia beetles in *C. canadensis* deployed in Virginia.

Treatment	Mean (± SE) Number of			
	Eggs Recovered per Tree	Larvae Recovered per Tree	Pupae Recovered per Tree	*X. germanus* Recovered per Tree
Flooded	0.0 ± 0.0	2.5 ± 1.5 ^a^	1.0 ± 1.0	0.5 ± 0.3 ^a^
Flooded plus Azoxystrobin	0.0 ± 0.0	0.0 ± 0.0	0.0 ± 0.0	0.3 ± 0.3 ^a^
Flooded plus Potassium Phosphite	0.0 ± 0.0	1.0 ± 0.6 ^a^	0.0 ± 0.0	1.3 ± 0.9 ^a^
Non-Flooded	0.0 ± 0.0	0.0 ± 0.0	0.0 ± 0.0	0.0 ± 0.0
Non-Flooded plus Azoxystrobin	0.0 ± 0.0	0.0 ± 0.0	0.0 ± 0.0	0.0 ± 0.0
Non-Flooded plus Potassium Phosphite	0.0 ± 0.0	0.0 ± 0.0	0.0 ± 0.0	0.0 ± 0.0
*t*; *P*: Flooded vs. Flooded plus Azoxystrobin	–	–	–	−0.57; 0.57
*t*; *P*: Flooded vs. Flooded plus Potassium Phosphite	–	−1.55; 0.12	–	1.10; 0.27

Means with different letters within a column are significantly different using a count regression analysis with a non-zero inflated model and Poisson distribution (df = 1; *n* = 4 trees per treatment). Only the flooded trees were included as part of the analysis because no attacks occurred on any of the non-flooded trees. Treatments with no eggs, larvae, or pupae were not included in the analyses.
